# Primary prevention of HPV-related diseases from the patients’ perspective in Poland

**DOI:** 10.1097/CEJ.0000000000000866

**Published:** 2023-11-30

**Authors:** Dominika Trojnarska, Robert Jach

**Affiliations:** a Faculty of Health Sciences, Jagiellonian University Medical College; b Faculty of Medicine, Jagiellonian University Medical College, Kraków, Poland

**Keywords:** cervical cancer, human papillomavirus, patients’ knowledge, prevention, vaccination

## Abstract

**Objectives:**

This study aimed to assess the knowledge of human papillomavirus (HPV) and attitudes toward HPV vaccination (HPVv) among female patients in Poland, investigating the impact of sociodemographic factors on these aspects. The study also explored awareness of state-funded bivalent vaccination and gauged willingness to vaccinate children, especially in the aspect of the newly introduced nonavalent vaccine.

**Material and methods:**

An 11-question questionnaire was administered to newly referred patients at a dysplasia consultation center in Kraków University Hospital between February and December 2022. Statistical analysis using IBM SPSS Statistics 25 evaluated sociodemographic characteristics, HPV knowledge, attitudes toward HPVv and correlations among responses.

**Results:**

By December 2022, 187 completed forms were received, primarily from women aged 30-40 years, residing in large cities, and with higher education qualifications. While most were aware of HPV’s association with cancer and abnormal cytology, over 40% were unaware of its asymptomatic nature. Higher education is correlated with better HPV awareness. Participants generally showed positive attitudes toward HPVv for themselves and their children, yet only a small fraction had received the vaccine. Education significantly influenced HPV knowledge, with higher education levels linked to better awareness and willingness to vaccinate children. Awareness of HPV is positively correlated with knowledge test performance and vaccination attitudes.

**Conclusion:**

The study revealed a lack of awareness regarding government co-financing for the bivalent vaccine. Campaigns endorsing reimbursed vaccination were found to be inadequate, highlighting the need for corrective measures to enhance awareness and improve vaccination rates, particularly for individuals outside the age range between 12 and 13 years relying on self-financing or sporadic government initiatives.

## Introduction

Globally, anogenital human papillomavirus (HPV) is the most common sexually transmitted infection that can cause different diseases in both males and females. Persistent infection with high-risk HPV may lead to high-grade lesions and, if undetected and untreated, to cancer. HPV has been identified as the causal factor in over 90% of cervical cancer cases, 75% of vaginal malignancies, and almost 70% of vulvar cancers (CDC, 2023). These conditions were previously considered unpreventable prior to the introduction of HPV vaccination (HPVv). The recognition of the link between HPV infection (HPVi) and various benign and malignant conditions, coupled with the development of preventive HPVv, has brought about groundbreaking changes in the prevention of the aforementioned health issues (Spayne and Hesketh, 2021).

Primary prevention of HPV-related diseases became available in 2006 with the licensing of the quadrivalent vaccine, followed by the bivalent vaccine in 2007 and the nonavalent vaccine in 2014 (Akhatova *et al*., 2022). All three vaccines offer protection against HPV types 16 and 18, with the quadrivalent vaccine additionally covering types 6 and 11, and the nonavalent vaccine extending protection to types 31, 33, 45, 52, and 58. These vaccines are suitable for both men and women starting at 9 years of age and have demonstrated effectiveness in preventing HPVi and associated neoplastic conditions (CDC, 2021; Issanov *et al*, 2022). To date, approximately 125 countries have incorporated HPVv into their immunization programs, and according to the WHO, 21% of women globally have received at least one dose of a prophylactic HPVv (Human Papillomavirus, 2023).

While the successful implementation of HPVv campaigns in countries like the USA, UK, Australia, and Argentina has led to a significant reduction in the incidence and prevalence of HPV-related cancers, it is important to note that the outcomes of these campaigns vary on a global scale (Karafillakis *et al*., 2019; Aimagambetova and Azizan, 2022). Several factors contribute to this disparity, including limited public knowledge and awareness about HPV and its associated conditions, the absence of comprehensive educational initiatives, and deficiencies in the implementation of vaccination programs (Karafillakis *et al*., 2019).

Poland is home to approximately 16.9 million women aged 15 and older who face the risk of developing cervical cancer. According to data from Center of HPV and Cancer, current statistics reveal that each year, 3862 women receive a diagnosis of cervical cancer, and 2137 lose their lives to this disease. Cervical cancer stands as the sixth most prevalent cancer among Polish women and ranks third among women aged 15–44. An estimated 3.4% of women in the general population are believed to carry cervical HPV-16/18 infection at any given time, and a significant 88.1% of invasive cervical cancers are linked to HPVs 16 or 18 (HPV INFORMATION CENTRE, 2023).

In the context of the coexistence of two arms of cervical cancer screening: publicly funded (in a structured and opportunistic model) and privately funded (exclusively in an opportunistic model), in 2022 the Polish Society of Colposcopy and Cervical Pathology introduced the standardization of the screening schedule for cervical cancer (Stanowisko PTKiPSM, 2023). The Society recommends using only clinically validated screening tools of proven efficacy and safety for the study population: the test for highly oncogenic HPV types, the HPV test performed at the same time as cytology (cotesting), gynecological cytology in preparation on a liquid medium (LBC) or conventional. The age of 25 is accepted as the starting age, and the age of 74 is considered the minimum age for ending screening.

As of primary prevention, starting from November 2021, one of the HPVv has been made available in Poland with a 50% reimbursement option. It is recommended for both girls and boys aged 9 years and older, as well as individuals up to 26 years of age who have not received previous vaccinations. Additionally, vaccination should be considered for individuals aged 27–45 years who have not been previously vaccinated and may still benefit from vaccination (Szczepienia.Info, 2023). Commencing June 2023, both bivalent and nonavalent vaccines have been provided free of charge in Poland for all children aged 12–13 (Augustynowicz E. Szczepienia.Info, 2023). Previously, individuals willing to get vaccinated had to cover the full cost of the vaccine themselves or, if they were eligible, make use of sporadic local government prevention programs.

## Objectives

With the introduction of 50% reimbursement for the bivalent HPVv in October 2021, this study aims to assess the general knowledge of HPV, HPVi, and their associations with abnormal cytology results and cervical cancer risk among female patients in Poland. The study also seeks to gauge the potential demand for vaccination among respondents by evaluating their willingness to receive the vaccine for themselves and their children. Sociodemographic factors such as age, place of residence, and education are analyzed to uncover potential variations in HPV knowledge and vaccination attitudes.

The study further was designed to explore correlations within respondents’ answers to identify factors influencing HPV knowledge and attitudes toward HPVv. Specifically, it investigates whether sociodemographic variables influence responses to questions related to HPV and HPVv. Additionally, the study examines whether general awareness of HPV’s existence translated into more detailed knowledge about the virus. Furthermore, the research explores whether mothers’ willingness to receive the HPVv is linked to their inclination to have their children vaccinated.

The HPV Vaccination Program, initiated in Poland in June 2023, provides free vaccination for girls and boys aged 12 and 13. However, individuals falling outside this age group are required to cover the vaccine costs themselves or rely on occasional local government prevention programs. Consequently, the availability of the bivalent vaccine with a 50% reimbursement presents an attractive option for those interested in vaccination. Unfortunately, many individuals appear to be unaware of this cost-saving opportunity, leading to a potential reduction in the overall vaccination rate.

The ultimate objective of this study is to raise awareness among healthcare professionals about the importance of discussing primary HPV prevention with their patients. Additionally, it highlights the need for increased government efforts to educate citizens about HPVi, which is one of the most common sexually transmitted diseases (STD).

## Material and methods

### Study subject and study design

An anonymous survey was conducted between February and December 2022, targeting newly referred patients to the dysplasia consultation center at the University Hospital in Kraków, Poland.

The study aimed to assess patients’ general knowledge of HPV, attitudes toward primary prevention, awareness of the 50% co-financing for HPVv, and sociodemographic characteristics. The authors wanted to examine various correlations among respondents’ knowledge and attitudes regarding HPV and the HPVv. Sociodemographic factors such as age, place of residence, and education were considered to assess their impact on participants’ responses. Additionally, the study explored factors influencing the willingness to get vaccinated and to vaccinate children. The analysis focused on identifying significant relationships between awareness of HPV, knowledge about HPV and the HPVv, and the willingness to get vaccinated.

Initially, the goal was to recruit 125 participants quickly, but due to a positive response, the target was extended. By the end of 2022, 187 questionnaires were collected. The survey was temporarily suspended from July to August 2022 during a holiday break when the main investigator was unavailable.

### Study variables

#### Independent variables

Independent variables were age, place of residence, and education. According to age, women were categorized into five major groups: younger than 30 years old, 30–40 years old, 41–50 years old, 51–60 years old, and older than 60 years old. They could choose between three different places of residence: a city with over 100 000 inhabitants, a city with fewer than 100 000 inhabitants, and a village. There were five levels of education: primary education, lower secondary education, basic vocational education, secondary education, and higher education.

#### Outcome variables

The attitude of the participants was reflected in their responses to questions related to HPV. These questions covered various aspects, including general knowledge about the existence of HPV, the link between HPV and cervical cancer, the symptoms of HPVi, HPV as an STD, the connection between HPV and abnormal cytology results, their willingness to undergo HPVv, their willingness to allow their children to get vaccinated, and their awareness of the 50% co-financing of the vaccine.

### Exclusion and inclusion criteria

Women presenting for the first time at the center with abnormal cytology results or other genital changes (e.g. cervical polyps, lichen sclerosus, precancerous lesions of the vagina and vulva) were included in the study. Exclusion criteria comprised patients under 18 years of age, those lacking consent for participation, individuals not proficient in the Polish language, and those with follow-up appointments (due to prior HPV and HPVv information obtained from the consulting physician).

The study featured a highly selective group of female respondents primarily referred to the dysplasia consultation center due to genital lesions, primarily attributed to HPV. This selectivity suggests that this group likely possessed more extensive knowledge of HPV than the general population. However, the inclusion of patients with HPV-independent cervical, vaginal, and vulvar lesions enriched the diversity of the study group. The questions were designed to be as simple as possible, which led to some ambiguity, especially in the question concerning the symptoms of HPV. HPVi may be asymptomatic, with the detection of HPV requiring a special test, or symptomatic in the case of condylomata acuminata. Additionally, in the authors’ opinion, HPV is sometimes mistaken for HBV in the Polish language.

### Study instrument

The survey consisted of questions designed by the authors, available in Polish (Appendix A, Supplemental digital content 1, http://links.lww.com/EJCP/A427). For the purpose of this manuscript, the English translation is provided in Appendix B, Supplemental digital content 2, http://links.lww.com/EJCP/A428. This questionnaire included 11 single-choice questions grouped into categories: basic HPV knowledge, attitudes toward HPVv with awareness of the 50% co-financing for vaccination, and respondents’ demographics (age, place of residence, education). The survey was accompanied by introductory information explaining the study’s purpose and providing contact details for the authors. Participants were informed that their participation was anonymous and voluntary. The inquiry covered topics that are part of general knowledge and should be typically discussed in healthcare settings, schools, and media.

Respondents were asked whether they had ever heard of the term HPV. A positive response indicated awareness of HPV, while a negative response indicated a lack of it. Specific questions about HPV were related to its potential to cause abnormal cervical cytology results and its association with cervical cancer. A ‘Yes’ response signified recognition of these associations, a ‘No’ response implied disbelief in them, and ‘I don’t know’ suggested uncertainty regarding the connections. Regarding the question of whether HPVi is asymptomatic, a ‘Yes’ response signified awareness that HPVis can be asymptomatic, a ‘No’ response indicated a belief that HPVi have symptoms, and ‘I don’t know’ suggested uncertainty about the symptomatology. Patients were tested on their knowledge of HPV as an STD, with a ‘Yes’ response indicating the correct answer, a ‘No’ response implying a belief that HPV is not an STD, and a third option suggesting uncertainty regarding the mode of transmission.

The second part of the questionnaire focused on women’s attitudes toward HPVv. Willingness to get vaccinated was assessed with a ‘Yes’ answer indicating willingness, ‘No’ indicating reluctance, and a third option for those already vaccinated. Responses assessing readiness to vaccinate offspring were based on preferences for vaccinating daughters, sons, both, or neither. The answer ‘My child/children have already been vaccinated’ indicated prior vaccination. The authors also aimed to determine the prevalence of patient awareness regarding the 50% reimbursement of one of the HPVv, using both affirmative and negative statements.

The survey concluded with three questions concerning sociodemographic data. Respondents could choose from five different age groups, starting with women younger than 30 years, followed by 30–40 years old, 41–50 years old, 51–60 years old and ending with patients over 60. The place of residence options included big cities with over 100 000 inhabitants, towns with up to 100 000 inhabitants, and villages. The education level was assessed using the five educational levels available in Poland: primary education, lower secondary education, basic vocational education, secondary education, higher education.

### Statistical analysis

Statistical analyses were conducted using IBM SPSS Statistics 25. These analyses encompassed frequency assessments, Spearman’s rho rank correlation analyses, χ² tests, and Fisher’s exact tests. A significance level of α = 0.05 was employed, with probability results within the range of 0.05 < *P* < 0.1 interpreted as significant at the level of the statistical trend.

### Ethical considerations

This research was conducted in compliance with the Declaration of Helsinki and received approval from the Jagiellonian University Medical College’s bioethics committee (No. 1072.6120.43.2022, dated 23 February 2022). From an ethical standpoint, no doubts were raised about the conduct of the study. Prior to being enrolled, prospective participants were provided with detailed information regarding the study’s objectives, methodologies, potential risks, and anticipated benefits. Subsequently, participants provided verbal consent upon understanding the voluntary and anonymous nature of the research.

## Results

### Sociodemographic characteristics of the respondents

A total of 187 women participated in the study. The sociodemographic characteristics of the respondents, including age, place of residence, and education, are presented in Table 1.

**Table 1:** Sociodemographic characteristics of the respondents

Age		<30 years old	30–40 years old	41–50 years old	51–60 years old	>60 years old
	*N*	40	83	24	23	17
	%	21.4	44.4	12.8	12.3	9.1
Place of residence		City >100 000 inhabitants	City <100 000 inhabitants	Village	No data available	
	*N*	104	34	48	1	
	%	55.6	18.2	25.7	0.5	
Education		Primary education	Lower secondary education	Basic vocational education	Secondary education	Higher education
	*N*	1	1	22	55	108
	%	0.5	0.5	11.8	29.4	57.8

In the study population, the most common age group was 30–40 years, with 83 respondents (44.4%). There were 40 respondents under 30 years of age (21.4%), 24 respondents aged 41–50 years (12.8%), 23 respondents aged 51–60 years (12.3%), and only 17 respondents (9.1%) were over 60 years of age.

A total of 186 people provided answers to the question about their place of residence. One data point was missing (0.5%). Within the sample, the majority of respondents resided in large cities with more than 100 000 inhabitants (104 people, 55.6%), 48 respondents lived in rural areas (25.7%), and 34 resided in small towns with up to 100 000 inhabitants (18.2%).

The final demographic variable considered in the study was the educational level of the surveyed women. The sample was primarily composed of women with tertiary education (108, 57.8%), followed by 55 respondents with secondary education (29.4%), 22 with vocational education (11.8%), and one person each with primary or lower secondary education (0.5%).

### HPV knowledge test

The study examined how the surveyed women responded to specific questions about HPV. The answers are presented in Table 2.

**Table 2:** Responses to the HPV knowledge test questions

		Yes	No	I do not know
Question 1: Have you heard of the human papillomavirus (HPV)?	*N*	151	36	-
	%	80.7	19.3	-
Question 2: Do you believe that HPV causes cervical cancer?	*N*	129	2	56
	%	69.0	1.1	29.9
Question 3: Is HPV infection asymptomatic?	*N*	84	24	79
	%	44.9	12.8	42.2
Question 4: Is HPV infection a sexually transmitted disease?	*N*	128	12	46
	%	68.8	6,5	24.7
Question 5: Can HPV infection cause an abnormal cytology result?	*N*	140	4	43
	%	74.9	2.1	23.0

The majority of respondents have already heard about HPV (80.7%). Most of them believe that the virus may lead to cervical cancer (69.0%) but at the same time, almost 30% were not aware of that fact. Only 2 women did not associate HPV with that type of cancer. Responses to the question of whether HPVi is asymptomatic were relatively diverse. 84 women believed that HPV gives symptoms. A similar number of respondents, 79, did not know the answer to this question. The infection was considered asymptomatic by 24 women. In terms of HPV as an STD, around 70% of the respondents correctly categorized this infection and almost 25% did not know the answer to this question. Just over 6% (12 surveyed women) did not consider HPV to be an STD. The final question on the HPV knowledge test focused on the connection between the virus and abnormal cytology results. The vast majority, nearly 75%, were aware that HPV may lead to abnormal cytology results. About 23% were not aware of this fact, and 4 respondents answered in the negative.

### HPVv attitude and knowledge test

The final part of the survey was questions about the HPVv, the answers to which are presented in Table 3.

**Table 3:** Responses to the HPVv attitude and knowledge test questions

		Yes	No	I have already been vaccinated	No data available	
Question 6: Are you willing to undergo HPV vaccination, which can protect against HPV infection?	*N*	152	27	6	2	
	%	81.3	14.4	3.2	1.1	

		Yes, but only my daughter(s)	Yes, but only my son(s)	Yes, regardless of the sex of my child/children	My child/children have already been vaccinated	No
Question 7: Are you willing to have your child/ children vaccinated against HPV?	*N*	40	5	113	4	22
	%	21.4	2.7	60.4	2.1	11.8

		Yes	No			
Question 8: Are you aware that the HPV vaccine is 50% co-funded?	*N*	32	155			
	%	17.1	82.9			

Respondents displayed a strong willingness to receive the HPVv, with 152 (81.3%) women expressing their willingness, in addition to the 6 (3.2%) who had been vaccinated, indicating a highly positive attitude towards vaccination: a total of 158 (84.5%) women would like to be vaccinated or already are. 27 respondents answered negatively, while two respondents did not provide an answer to this question at all.

When asked about vaccinating their offspring, affirmative answers once again prevailed. 113 respondents indicated their readiness to vaccinate their children regardless of gender (60.4%), while 40 (21.4%) expressed a willingness to vaccinate only their daughters. An additional five women (2.7%) were prepared to vaccinate only their male children. In four cases (2.1%), the response was ‘My child has already been vaccinated’. These results demonstrate a remarkably high willingness among mothers to vaccinate their offspring, with a total of 162 mothers (86.6%) either desiring or already having vaccinated their children. Twenty-two respondents provided a negative response (11.8%), and three did not answer this question.

The final question inquired whether the women surveyed were aware of the 50% reimbursement available for the HPV vaccine. Only 32 out of 187 (17.1%) were aware of the opportunity to obtain the vaccine at a reduced cost, while the remaining 155 (82.9%) were not aware of this option.

### Correlations between sociodemographic factors and knowledge about HPV/HPVv, as well as attitudes toward HPVv

The authors decided to analyze selected correlations between the answers given by the respondents in order to find out which factors determine knowledge about HPV and attitudes towards the HPVv. It was investigated whether sociodemographic factors, including age, place of residence, and education, had an impact on responses to the HPV and HPVv knowledge and attitude test. In addition, factors influencing willingness to get vaccinated and willingness to vaccinate their children were examined. The detailed results of the statistical analysis are presented in Appendix C, Supplemental digital content 3, http://links.lww.com/EJCP/A429.

### Age

We conducted an analysis to assess whether age had an impact on HPV awareness. A correlation analysis was performed, but it did not reveal a significant relationship between these variables (*P* = 0.251). Thus, no influence of the respondents’ age on the awareness of HPV’s existence was demonstrated. The distribution of responses can be found in Table 4 in Appendix C, Supplemental digital content 3, http://links.lww.com/EJCP/A429.

Subsequently, we investigated whether women of different age groups provided different responses to more specific questions in the HPV knowledge test. As presented in Table 5 in Appendix C, Supplemental digital content 3, http://links.lww.com/EJCP/A429, two statistically significant results were observed. Firstly, with increasing age, the proportion of ‘Don’t know’ responses to the question of whether HPVi is asymptomatic increased. Secondly, the proportion of negative responses to the question of whether HPVi can cause an abnormal cytology result was also higher. A result at the level of statistical trend was noted for the question about whether HPV is an STD. The 51–60 and >60 age groups were significantly less likely to provide affirmative answers compared to the other three age groups, where the proportion of affirmative answers was similar. Regarding knowledge about whether HPV causes cervical cancer, no significant relationship was found, even at the level of statistical trend.

We also examined whether the age of the mothers influenced their willingness to vaccinate themselves and their children. This analysis revealed one statistically significant relationship, as shown in Table 6 in Appendix C, Supplemental digital content 3, http://links.lww.com/EJCP/A429. As age increased, the percentage of those willing to vaccinate their child regardless of gender decreased, while the percentage of those willing to vaccinate only their daughter increased. In the case of willingness to vaccinate oneself against HPV, there was no statistically significant correlation. However, it is worth noting that the group of individuals aged 60 and older stood out. It is advisable to further investigate their attitudes with a larger study sample.

Furthermore, we verified whether the age of the subjects was associated with awareness of the 50% reimbursement for the HPV vaccine, but no significant result was recorded, even at the level of statistical trend (*P* = 0.513). The distribution of responses is summarized in Table 7 in Appendix C, Supplemental digital content 3, http://links.lww.com/EJCP/A429.

### Place of residence

An investigation was conducted to assess the potential influence of respondents’ place of residence on their responses regarding HPV knowledge and attitudes toward the HPVv. Initially, we analyzed whether place of residence correlated with HPV awareness; however, no statistically significant relationship was found between these variables (*P* = 0.704). Detailed response distributions are provided in Table 8 in Appendix C, Supplemental digital content 3, http://links.lww.com/EJCP/A429.

Subsequently, we examined whether place of residence was associated with the respondents’ knowledge level. As indicated in Table 9 in Appendix C, Supplemental digital content 3, http://links.lww.com/EJCP/A429, no statistically significant results were observed. Place of residence did not show a significant association with the level of knowledge concerning HPV.

Table 10 in Appendix C, Supplemental digital content 3, http://links.lww.com/EJCP/A429 summarizes the subjects’ place of residence and their willingness to vaccinate themselves and their children. Once again, the results did not approach statistical significance.

We also investigated whether the place of residence of the subjects was linked to their awareness of the 50% reimbursement for the HPVv. However, no significant relationship was established between these variables (*P* = 0.891). Further details on response distributions can be found in Table 11 in Appendix C, Supplemental digital content 3, http://links.lww.com/EJCP/A429.

### Education

In the subsequent analysis, we explored the potential relationship between the respondents’ educational level and their responses regarding HPV knowledge and attitudes toward HPVv. Due to the limited number of individuals with primary and junior high school education (one respondent each), they were excluded from the analysis to ensure the reliability of our findings.

First, we investigated whether the level of education was associated with HPV awareness. The analysis revealed a statistically significant result (*P* < 0.001). As depicted in Table 12 in Appendix C, Supplemental digital content 3, http://links.lww.com/EJCP/A429, individuals with higher education exhibited the highest proportion of affirmative responses.

We then assessed whether the educational level of the subjects was linked to their knowledge level. As shown in Table 13 of Appendix C, Supplemental digital content 3, http://links.lww.com/EJCP/A429 three statistically significant results were observed. Respondents with higher education were more likely to provide affirmative answers to questions concerning whether HPV causes cervical cancer, whether HPVi is asymptomatic, and whether HPV is sexually transmitted. Regarding the question related to HPV as an STD, no significant relationship was found, even at the level of statistical trend.

Table 14 in Appendix C, Supplemental digital content 3, http://links.lww.com/EJCP/A429 presents a comparison between the subjects’ educational level and their willingness to vaccinate themselves and their child/children. A statistically significant relationship was observed concerning the willingness to vaccinate one’s own child: With an increase in the level of education, the percentage of individuals willing to vaccinate their child/children, regardless of gender, also increased. Conversely, the percentage of respondents providing a negative answer or expressing willingness to vaccinate only a female child decreased. However, with regard to the willingness to vaccinate oneself, no significant results were obtained, even at the level of statistical trend.

Finally, we examined whether the educational level of the subjects was associated with their awareness of the 50% reimbursement for the HPVv. No significant relationship was found between these variables (*P* = 0.508). The distribution of responses is summarized in Table 15 in Appendix C, Supplemental digital content 3, http://links.lww.com/EJCP/A429.

### Correlations between HPV awareness, knowledge and attitudes toward HPVv

We explored potential statistically significant correlations among respondents’ awareness of HPV existence, their knowledge about HPV and HPVv, as well as their willingness to get vaccinated themselves and vaccinate their children. Finally, we analyzed the knowledge of the 50% reimbursement of HPVv in relation to HPV awareness.

An analysis was conducted to assess whether participants’ awareness of HPV was related to their level of knowledge about the virus. The results are presented in Table 16 of Appendix C, Supplemental digital content 3, http://links.lww.com/EJCP/A429. All four results were statistically significant. Those aware of HPV were more likely to provide affirmative answers and less likely to indicate ‘don’t know’ responses. Interestingly, the differences in the proportion of ‘no’ answers between the compared groups were relatively small.

We conducted an analysis to explore potential correlations between the awareness of HPV and the willingness of participants to vaccinate themselves and their children against HPV. The results are summarized in Table 16 in Appendix C, Supplemental digital content 3, http://links.lww.com/EJCP/A429. A statistically significant relationship was observed for the willingness to vaccinate one’s child. Specifically, individuals aware of HPV were more likely to express a willingness to vaccinate their child regardless of gender and less likely to provide a negative response. Furthermore, among those aware of HPV, there was presence of individuals who had already vaccinated their child. However, no significant results were recorded regarding the willingness to vaccinate oneself, even at the level of statistical trend.

Table 19 in Appendix C, Supplemental digital content 3, http://links.lww.com/EJCP/A429 summarizes the willingness to vaccinate oneself and one’s child. A statistically significant relationship was noted. Individuals who had already been vaccinated themselves were unequivocally ready, or had already vaccinated their child. The proportion of respondents indicating a readiness to vaccinate their child, while still lower than those willing to vaccinate themselves, was significantly higher compared to those who did not declare a willingness to vaccinate themselves.

In Table 19 of Appendix C, Supplemental digital content 3, http://links.lww.com/EJCP/A429, we examined whether the willingness to vaccinate one’s child was related to the participants’ level of knowledge about HPV. All four results were statistically significant. Those willing to vaccinate their child regardless of gender were more likely to provide affirmative answers and less likely to respond with ‘I don’t know’. Conversely, those willing to vaccinate their child based on gender had intermediate results, while those not willing to vaccinate their child most frequently provided affirmative answers and ‘I don’t know’ responses.

## Discussion

To date, there have been no published studies investigating attitudes toward the HPVv among Polish female patients. Thus, this is the first study that aims to examine HPV knowledge and attitudes toward the HPVv and to explore factors associated with a positive attitude toward the vaccine in this group of women. Poland has some of the highest incidence and mortality rates from cervical malignancies in Europe. Up to 40% of newly diagnosed cases of cervical cancer are in an advanced stage. According to National Cancer Registry (KRN), barely 27% of Polish women undergo cytological examinations of the cervix (Krajowy Rejestr Nowotworów [Internet] 2021). However, this data is biased because many patients consult gynecologists in private practices, which are not obliged to keep statistics on secondary prevention of cervical cancer. Due to the abovementioned data, primary prevention of cervical malignancies in Poland is of utmost importance.

Almost 81% of surveyed women were aware of the existence of HPV, which is comparable to the results of a recent study conducted in September 2022 among parents in Poland, where just over 74% were familiar with the term HPV (Sobierajski *et al*, 2023). In a study involving 2272 women attending gynecological clinics, it was observed that 46% of those who were already aware of HPV were aware of its association with cervical cancer (Issa *et al*., 2021). In our research group, the level of awareness surpassed this figure, reaching an impressive 83%. We discovered that our respondents’ attitude towards HPVv was highly favorable. Nearly 85% expressed willingness to receive the vaccine or had already been vaccinated. Additionally, mothers showed a strong inclination to vaccinate their children, over 86% displayed a positive disposition towards primary prevention. Prior research has indicated that attitudes and intentions regarding HPVv can range from 15 to 95% across different societies, influenced by a multitude of factors (Khatiwada *et al*, 2021; Sallam *et al*., 2021; Aimagambetova *et al*, 2022). Unlike other studies, we did not find an association between the place of residence and mothers’ attitudes toward HPVv (Babi *et al*., 2023). The most influential sociodemographic factor affecting HPV knowledge and decision-making regarding HPVv was education. Women with higher educational backgrounds demonstrated significantly greater awareness of HPV and performed better on the knowledge assessment. They exhibited a willingness to vaccinate their children, irrespective of gender. Lower education level is the variable that most interferes with knowledge about HPV (Kops *et al*., 2019; Sobierajski *et al*, 2023). A similar study conducted in Kazakhstan found that the highest proportion of favorable attitudes was among women with university degrees, while the lowest was among those with only a high school education or incomplete high school education (Aimagambetova *et al*, 2022). The cited study reported a positive attitude towards HPVv, particularly among the youngest age group, and the lowest level of acceptance among the oldest age group. Regarding age, in our survey older participants were less informed about whether HPVi were asymptomatic or related to abnormal cytology results. Individuals over 50 years old were more likely to not recognize HPV as an STD. Furthermore, as age increased, fewer women expressed a willingness to vaccinate their children regardless of gender. In a 2019 Canadian study, it was found that the majority of participants held moderate to strong beliefs regarding the significance of HPVv as a crucial component of disease prevention. However, a significant number of patients harbored concerns about the safety of these vaccines (Steben *et al*, 2019).

Healthcare professionals cite financial concerns and parental attitudes and concerns as barriers to providing the HPVv to patients (Trojnarska *et al*, 2022). Presented results let us assume that adults are more eager to vaccinate themselves even if they do not know the pathogen but are more restrictive when it comes to vaccination of their children. It was evident that the more the patient was open to vaccination, the more she was willing to vaccinate her offspring. Parents often report needing more information before vaccinating their children. Concerns about the vaccine’s effect on sexual behavior, low perceived risk of HPVi, social influences, irregular preventive care, and vaccine cost were also identified as potential barriers among parents (Holman *et al*, 2014; Steben *et al*, 2019). Some parents of sons reported not vaccinating their sons because of the perceived lack of direct benefit (Sobierajski *et al*, 2023). Parents consistently cite healthcare professional recommendations as one of the most important factors in their decision to vaccinate their children (Holman *et al*, 2014; Sobierajski *et al*, 2023). A questionnaire about knowledge, attitudes and awareness of the HPV and HPVv was conducted among Polish gynecologists between January and May 2021, before the introduction of 50% reimbursement of the bivalent vaccine and long before implementation of a gender-neutral HPV immunization program in June 2023, showed that physicians’ general knowledge about HPV was good, independent of gender and age (Trojnarska *et al*, 2022). The acceptance of all vaccines was high, but the low availability of the HPVv seemed at that point to be the biggest problem stopping patients from getting them. Where children are concerned, information on health-seeking behavior is expected to be also provided by teachers. Unfortunately, it has previously been proven that teachers’ level of knowledge regarding HPV and its vaccination is lacking. Furthermore, researchers observed deficiency and mistakes in teachers’ attitude and behaviors towards HPV and its vaccine. The knowledge deficiency of male teachers about HPV and vaccination compared with female teachers was proven to be remarkable (Keten *et al*, 2021).

Only in the group with knowledge of HPV existence were there women who had already been vaccinated, as well as women who had vaccinated their children – regrettably, the numbers were low, with only 6 vaccinated patients and 4 vaccinated children. Importantly, all 4 vaccinated children had mothers who were either willing to get vaccinated or were already vaccinated themselves. Most of those surveyed (82.9%) were unaware of the 50% reimbursement for the HPVv. Few patients who knew about the reimbursement had also already heard about HPV. This lack of knowledge was alarming, even in the youngest and thus the most important surveyed subgroup. Even in the group of highly educated patients most of the women were not aware of the reimbursement. Earlier research showed that the primary sources of information about HPVv were television ads (61%), healthcare providers (52%), and friends (45%) (Unger *et al*, 2015). Other authors have found that the internet was the most commonly utilized source and was the only source used by a majority (80.9%) of information seekers. The next most frequently utilized sources, used by about 38% of cases each, were doctors and healthcare providers and brochures and pamphlets At the same time doctors and healthcare providers turned out to be the most trusted (Inglehart *et al*., 2016 Dec). Those results show how important the media and health professionals are in spreading awareness of available vaccinations.

Our study marks one of the initial attempts to asses HPV knowledge and vaccination attitudes among Polish women. Nonetheless, our focus solely on patients referred to the dysplasia consultation center means that our findings may not fully represent the broader female population in the country. The relatively small sample size underscores the need for expanding this study beyond its current pilot phase. Furthermore, future research should delve deeper into the attitudes of parents and especially mothers, who often serve as primary decision-makers when vaccination targeting children considered. These limitations provide valuable insights for future studies, where we aim to address them comprehensively, yielding a more accurate understanding of HPV knowledge and attitudes towards HPVV among Polish society.

To prevent cervical cancer, the WHO recommends targeting HPVv primarily in girls aged 9–14 years, ideally before they become sexually active. It is crucial to ensure high vaccination coverage right from the start. When coverage exceeds 80% in girls, it also lowers the risk of HPVi in boys. Vaccination of secondary target groups, such as females aged 15 years and older, boys, older males, or men who have sex with men, is advisable only if it is practical, cost-effective, and doesn’t divert resources away from vaccinating the primary target population or from effective cervical cancer screening programs (WHO 2022).

One of the barriers that has hindered access to the HPVv in Poland was its cost. We believe this issue remains relevant, which makes the 50% reimbursement of the bivalent vaccine an opportunity for individuals beyond the limited group of children aged 12–13 years covered by full reimbursement. A special group eligible for vaccination includes women after surgical excision of cervical lesions where the vaccination is given as prevention of recurrent disease - defined by the presence of at least one negative clinical evaluation between surgical treatment and the diagnosis of new cervical intraepithelial neoplasia in the form of cervical intraepithelial neoplasia 2+ (CIN 2+). Women who had previously undergone surgery for HPV-related lesions and got an adjuvant vaccine are at significantly reduced risk of subsequent HPV-related disease (Joura *et al*., 2012; Jentschke *et al*, 2020). However, a more recent meta-analysis showed that adjuvant vaccination does not impact the risk of having persistent disease defined as the detection of high-grade cervical lesions at the time of first follow-up visit, especially in patients with positive margins (Di Donato *et al*., 2021). The therapeutic role of HPVv remains a subject of ongoing debate, and a definitive conclusion in this regard is currently lacking. A systematic review and meta-analysis conducted in 2022 shed light on this matter, revealing that early studies suggested a potential reduction in recurrence rates when HPVv were administered following a loop electrosurgical excision procedure. Nevertheless, subsequent trials have yielded conflicting evidence, suggesting that HPVv administration in such cases may not confer a substantial benefit (Eriksen *et al*, 2022 Jun). Large-scale, high-quality randomized controlled trials are necessary to determine both the effectiveness and cost-effectiveness of HPVv in women undergoing treatment for HPV-related diseases (Kechagias *et al*., 2022).

The consideration of persistent and recurrent HPVi becomes crucial in the context of the reproductive health of women who have undergone surgical treatment for CIN 2+ lesions. A systematic review conducted in 2020 evaluated the impact of excisional treatments on outcomes such as preterm delivery (PD), lower birth weight (LBW), preterm premature rupture of membranes (pPROM), and obstetric outcomes. The study found an increased risk of PD, LBW, and pPROM occurring before 37 weeks of pregnancy, particularly following cold-knife conization and large loop excision of the transformation zone procedures (Monti *et al*., 2021; Åström and Turkmen, 2023). In cases of high-grade squamous intraepithelial lesion persistence, especially after incomplete resection, margin involvement, or uncertain margin status, or recurrent disease, the consideration of re-excision underscores the importance of further investigating the potential benefits of adjuvant vaccination. It must be emphasized that vaccination against HPV as primary prevention of cervical cancer plays an important role in minimizing the necessity of treatment of pre-cancerous lesions and its complications. Early complications of surgical treatment include bleeding, in some cases requiring a second surgery and/or blood transfusion. Late complications are abovementioned obstetrical outcomes, with preterm birth being the most important.

The intricate communication hurdles surrounding HPVv and their resultant low vaccination rates can be effectively navigated through comprehensive community education campaigns. These initiatives should prioritize addressing parental concerns both pre- and post-implementation of HPVv programs while emphasizing the safety and efficacy of these vaccines for the prevention of HPV-related diseases in both genders. This approach is substantiated by multiple references. Sustained endeavors are warranted to disseminate transparent, evidence-based information, fostering increased vaccination coverage and, consequently, a reduction in HPV-related preinvasive and invasive conditions, in alignment with various sources.

### Conclusion

Our study indicates that while there is a relatively high level of awareness about HPV among the surveyed women, there are still knowledge gaps and misconceptions, particularly regarding symptoms and the availability of vaccine reimbursement. Education and increased awareness of available support for vaccination are essential strategies for improving HPVv. Additionally, the positive attitudes expressed toward both personal and child vaccination suggest a potential for higher immunization coverage if these awareness and knowledge gaps are addressed effectively.

## Acknowledgements

This survey could only be conducted thanks to continuing support of the Jagiellonian University Medical College (UJ CM) and the Polish Society of Colposcopy and Cervical Pathology (PTKiPSM).

### Conflicts of interest

There are no conflicts of interest.

## Supplementary Material






