# Supplementary material for: Primary prevention of HPV-related diseases from the patients’ perspective in Poland
Source: Eur J Cancer Prev. 2023 Nov 30;33(4):299–308. doi: 10.1097/CEJ.0000000000000866 (PMC11155277; doi:10.1097/CEJ.0000000000000866)
Supplement: Supplementary file 1 [file ejcp-33-299-s001.pdf]

APPENDIX A

Szanowna Pani,

Pod kierownictwem naukowym Profesora Roberta Jacha prowadzimy w Klinice Endokrynologii Ginekologicznej i Ginekologii Szpitala Uniwersyteckiego w Krakowie badanie ankietowe dotyczące wirusa brodawczaka ludzkiego (HPV). Wnioski płynące z danych zebranych dzięki Pani uprzejmości pozwolą nam na analizę wiedzy Pacjentek na temat profilaktyki pierwotnej raka szyjki macicy.

Ankieta jest anonimowa i składa się z 11 pytań zamkniętych. Jej wypełnienie zajmie Pani 2 minuty.

Wszelkie pytania oraz dodatkowe uwagi proszę kierować na adres mailowy: dtrojnarska@su.krakow.pl

Z góry dziękujemy za udział w badaniu ankietowym.

Z wyrazami szacunku

Prof. dr hab. med. Robert Jach

Dr n. med. Dominika Trojnarska, MHBA

Pytanie 1
Czy słyszała Pani o wirusie brodawczaka ludzkiego (HPV)?
☐ Tak
☐ Nie

Pytanie 2
Czy uważa Pani, że wirus HPV powoduje raka szyjki macicy?
☐ Tak
☐ Nie
☐ Nie wiem

Pytanie 3
Czy zakażenie wirusem HPV jest bezobjawowe?
☐ Tak

□ Nie
□ Nie wiem

Pytanie 4
Czy zakażenie HPV jest chorobą przenoszoną drogą płciową?
□ Tak
□ Nie
□ Nie wiem

Pytanie 5
Czy zakażenie HPV może spowodować nieprawidłowy wynik cytologii?
□ Tak
□ Nie
□ Nie wiem

Pytanie 6
Czy jest Pani skłonna poddać się szczepieniu przeciwko HPV, która może chronić przed zakażeniem HPV?
□ Tak
□ Nie
□ Jestem już zaszczepiona

Pytanie 7
Czy jest Pani skłonna poddać swoje dziecko / dzieci szczepieniu przeciwko HPV?
□ Tak, ale tylko moją córkę / córki
□ Tak, ale tylko mojego syna / synów
□ Tak, niezależnie od płci mojego dziecka / dzieci
□ Nie
□ Moje dziecko / dzieci są już zaszczepione
Pytanie 8
Czy wie Pani, że szczepionka przeciwko HPV jest w 50% refundowana?
□ Tak
□ Nie

Pytanie 9
Do której z podanych grup wiekowych Pani należy?
□ mniej niż 30 lat
□ 30-40 lat
□ 41-50 lat
□ 51-60 lat
□ powyżej 60 lat

Pytanie 10

W jakiej miejscowości Pani mieszka?

 miasto powyżej 100 tys. mieszkańców

 miasto do 100 tys. mieszkańców

 wieś

Pytanie 11

Jakie ma Pani wykształcenie?

 podstawowe

 gimnazjalne

 zasadnicze

 średnie

 wyższe.
