# Supplementary material for: Primary prevention of HPV-related diseases from the patients’ perspective in Poland
Source: Eur J Cancer Prev. 2023 Nov 30;33(4):299–308. doi: 10.1097/CEJ.0000000000000866 (PMC11155277; doi:10.1097/CEJ.0000000000000866)
Supplement: Supplementary file 2 [file ejcp-33-299-s002.pdf]

APPENDIX B

Dear Madam,

Under the scientific direction of Professor Robert Jach, we are conducting a survey on human papillomavirus (HPV) in the Department of Gynaecological Endocrinology and Gynaecology at the University Hospital in Kraków.
The conclusions from the data collected courtesy of you will allow us to analyse patients' knowledge of primary prevention of cervical cancer.

The survey is anonymous and consists of 11 closed questions. It will take you 2 minutes to complete.

Please send any questions or additional comments to the following e-mail address: dtrojnarska@su.krakow.pl

Thank you in advance for your participation in the survey.

Yours sincerely

Prof. dr hab. med. Robert Jach

Dr n. med. Dominika Trojnarska, MHBA

Question 1
Have you heard of the human papilloma virus (HPV)?
☐ Yes
☐ No

Question 2
Do you believe that HPV causes cervical cancer?
☐ Yes
☐ No
☐ I don't know

Question 3
Is HPV infection asymptomatic?
☐ Yes

□ No

□ I don’t know

Question 4

Is HPV infection a sexually transmitted disease?

□ Yes

□ No

□ I don’t know

Question 5

Can HPV infection cause an abnormal cytology result?

□ Yes

□ No

□ I don’t know

Question 6

Are you willing to undergo HPV vaccination, which can protect against HPV infection?

□ Yes

□ No

□ I have already been vaccinated

Question 7

Are you willing to have your child / children vaccinated against HPV?

□ Yes, but only my daughter(s)

□ Yes, but only my son(s)

□ Yes, regardless of the sex of my child / children

□ No

□ My child / children have already been vaccinated

Question 8

Are you aware that the HPV vaccine is in 50% co-funded?

□ Yes

□ No

Question 9

Which of the following age groups do you belong to?

□ less than 30 years old

□ 30-40 years old

□ 41-50 years old

□ 51-60 years old

□ over 60 years old

Question 10

Where do you live?

- city with more than 100,000 inhabitants
- city with up to 100,000 inhabitants
- village

Question 11

What is your educational background?

- primary education
- lower secondary education
- basic vocational education
- secondary education
- higher education.
