# Supplementary material for: Primary prevention of HPV-related diseases from the patients’ perspective in Poland
Source: Eur J Cancer Prev. 2023 Nov 30;33(4):299–308. doi: 10.1097/CEJ.0000000000000866 (PMC11155277; doi:10.1097/CEJ.0000000000000866)
Supplement: Supplementary file 3 [file ejcp-33-299-s003.pdf]

APPENDIX C

Tab. 4. Relationship between surveyed women's age and their awareness of HPV.

			Age				
			< 30 years old	30-40 years old	41-50 years old	51-60 years old	> 60 years old
Have you heard of the human papilloma virus (HPV)?	Yes	*N*	33	69	20	18	11
		%	82,50%	83,10%	83,30%	78,30%	64,70%
	No	*N*	7	14	4	5	6
		%	17,50%	16,90%	16,70%	21,70%	35,30%

Tab. 5. Correlation between surveyed women's age and their knowledge of HPV.

			Age					
			< 30 years old	30-40 years old	41-50 years old	51-60 years old	> 60 years old	
Do you believe that HPV causes cervical cancer?	Yes	*N*	28	60	17	15	9	*p* = 0,750
		%	70,00%	72,30%	70,80%	65,20%	52,90%	
	No	*N*	1	1	0	0	0	
		%	2,50%	1,20%	0,00%	0,00%	0,00%	
	I don’t know	*N*	11	22	7	8	8	
		%	27,50%	26,50%	29,20%	34,80%	47,10%	
Is HPV infection asymptomatic?	Yes	*N*	20	42	13	6	3	*p* = 0,007 *V* = 0,23
		%	50,00%	50,60%	54,20%	26,10%	17,60%	
	No	*N*	8	12	0	2	2	
		%	20,00%	14,50%	0,00%	8,70%	11,80%	
	I don’t know	*N*	12	29	11	15	12	
		%	30,00%	34,90%	45,80%	65,20%	70,60%	
Is HPV infection a sexually transmitted disease?	Yes	*N*	29	64	17	10	8	*p* = 0,056 *V* = 0,19
		%	72,50%	77,10%	70,80%	43,50%	50,00%	
	No	*N*	2	5	2	2	1	
		%	5,00%	6,00%	8,30%	8,70%	6,30%	
	I don’t know	*N*	9	14	5	11	7	
		%	22,50%	16,90%	20,80%	47,80%	43,80%	
Can HPV infection cause an abnormal cytology result?	Yes	*N*	31	63	21	14	11	*p* = 0,037 *V* = 0,21
		%	77,50%	75,90%	87,50%	60,90%	64,70%	
	No	*N*	0	0	1	1	2	
		%	0,00%	0,00%	4,20%	4,30%	11,80%	
	I don’t know	*N*	9	20	2	8	4	
		%	22,50%	24,10%	8,30%	34,80%	23,50%	

Tab. 6. Relationship between respondents' age and their attitudes towards HPVv.

			Age					
			< 30 years old	30-40 years old	41-50 years old	51-60 years old	> 60 years old	
Are you willing to undergo HPV vaccination, which can protect against HPV infection?	Yes	*N*	35	70	19	18	10	*p* = 0,110
		%	87,50%	85,40%	79,20%	81,80%	58,80%	
	No	*N*	4	9	3	4	7	
		%	10,00%	11,00%	12,50%	18,20%	41,20%	
	I have already been vaccinated	*N*	1	3	2	0	0	
		%	2,50%	3,70%	8,30%	0,00%	0,00%	
Are you willing to have your child / children vaccinated against HPV?	Yes, but only my daughter(s)	*N*	5	15	5	8	7	*p* < 0,001 *V* = 0,26
		%	12,50%	18,50%	20,80%	36,40%	41,20%	
	Yes, but only my son(s)	*N*	0	1	2	1	1	
		%	0,00%	1,20%	8,30%	4,50%	5,90%	
	Yes, regardless of the sex of my child / children	*N*	29	60	12	9	3	
		%	72,50%	74,10%	50,00%	40,90%	17,60%	
	no	*N*	6	5	2	3	6	
		%	15,00%	6,20%	8,30%	13,60%	35,30%	
	My child / children have already been vaccinated	*N*	0	0	3	1	0	
		%	0,00%	0,00%	12,50%	4,50%	0,00%	

Tab. 7. Correlation between respondents' age and their awareness of the 50% HPVv reimbursement.

			Age				
			< 30 years old	30-40 years old	41-50 years old	51-60 years old	> 60 years old
Are you aware that the HPV vaccine is in 50% co-funded?	Yes	*N*	4	15	6	5	2
		%	10,00%	18,10%	25,00%	21,70%	11,80%
	No	*N*	36	68	18	18	15
		%	90,00%	81,90%	75,00%	78,30%	88,20%

Tab. 8. Relationship between respondents' place of residence and their awareness of HPV.

			city > 100,000 residents	city < 100,000 residents	village
Have you heard of the human papilloma virus (HPV)?	yes	*N*	86	27	37
		%	82,70%	79,40%	77,10%

no	*N*	18	7	11
	%	17,30%	20,60%	22,90%

Tab. 9. Correlation between respondents' place of residence and their knowledge about HPV.

			City > 100,000 residents	City < 100,000 inhabitants	Village	
Do you believe that HPV causes cervical cancer?	Yes	*N*	74	23	31	p = 0,727
		%	71,20%	67,60%	64,60%	
	No	*N*	2	0	0	
		%	1,90%	0,00%	0,00%	
	I don't know	*N*	28	11	17	
		%	26,90%	32,40%	35,40%	
Is HPV infection asymptomatic?	Yes	*N*	50	17	16	$\chi^2(2)$ = 6,94 p = 0,139
		%	48,10%	50,00%	33,30%	
	No	*N*	10	3	11	
		%	9,60%	8,80%	22,90%	
	I don't know	*N*	44	14	21	
		%	42,30%	41,20%	43,80%	
Is HPV infection a sexually transmitted disease?	Yes	*N*	73	22	32	p = 0,237
		%	70,90%	64,70%	66,70%	
	No	*N*	7	0	5	
		%	6,80%	0,00%	10,40%	
	I don't know	*N*	23	12	11	
		%	22,30%	35,30%	22,90%	
Can HPV infection cause an abnormal cytology result?	Yes	*N*	75	25	39	p = 0,260
		%	72,10%	73,50%	81,30%	
	No	*N*	1	2	1	
		%	1,00%	5,90%	2,10%	
	I don't know	*N*	28	7	8	
		%	26,90%	20,60%	16,70%	

Tab. 10. Relationship between respondents' place of residence and their attitudes toward HPVv.

			city > 100,000 residents	city < 100,000 inhabitants	village	
Are you willing to undergo HPV vaccination, which can	yes	*N*	82	29	41	p = 0,811
		%	79,60%	85,30%	85,40%	
	no	*N*	16	5	6	

protect against HPV infection?		%	15,50%	14,70%	12,50%	
	I have already been vaccinated	N	5	0	1	
		%	4,90%	0,00%	2,10%	
Are you willing to have your child / children vaccinated against HPV?	Yes, but only my daughter(s)	N	19	7	13	p = 0,733
		%	18,80%	20,60%	27,10%	
	Yes, but only my son(s)	N	4	0	1	
		%	4,00%	0,00%	2,10%	
	Yes, regardless of the sex of my child / children	N	62	24	27	
		%	61,40%	70,60%	56,30%	
	no	N	12	3	7	
		%	11,90%	8,80%	14,60%	
	My child / children have already been vaccinated	N	4	0	0	
		%	4,00%	0,00%	0,00%	

Tab. 11. Correlation between respondents' place of residence and their awareness of the 50% HPVv reimbursement.

			City > 100,000 residents	City < 100 thousand inhabitants	Village
Are you aware that the HPV vaccine is in 50% co-funded?	Yes	*N*	18	5	9
		%	17,30%	14,70%	18,80%
	No	*N*	86	29	39
		%	82,70%	85,30%	81,30%

Tab. 12. Relationship between education levels of the surveyed women and their awareness of HPV.

			basic vocational education	secondary education	higher education
Have you heard of the human papilloma virus (HPV)?	yes	*N*	14	37	98
		%	63,60%	67,30%	90,70%
	no	*N*	8	18	10
		%	36,40%	32,70%	9,30%

Tab. 13. Correlation between respondents' education level and their knowledge of HPV.

			basic vocational education	secondary education	higher education	
Do you believe that HPV causes cervical cancer?	yes	*N*	12	27	88	p < 0,001<br>V = 0,24
		%	54,50%	49,10%	81,50%	

	no	*N*	0	1	1	
		%	0,00%	1,80%	0,90%	
	I don't know	*N*	10	27	19	
		%	45,50%	49,10%	17,60%	
Is HPV infection asymptomatic?	yes	*N*	4	18	61	$\chi^2(2) = 16{,}64$<br>$p = 0{,}002$<br>$V = 0{,}21$
		%	18,20%	32,70%	56,50%	
	no	*N*	3	8	13	
		%	13,60%	14,50%	12,00%	
	I don't know	*N*	15	29	34	
		%	68,20%	52,70%	31,50%	
Is HPV infection a sexually transmitted disease?	yes	*N*	13	31	83	$p = 0{,}025$<br>$V = 0{,}17$
		%	59,10%	56,40%	77,60%	
	no	*N*	1	4	7	
		%	4,50%	7,30%	6,50%	
	I don't know	*N*	8	20	17	
		%	36,40%	36,40%	15,90%	
Can HPV infection cause an abnormal cytology result?	yes	*N*	15	37	87	$p = 0{,}194$
		%	68,20%	67,30%	80,60%	
	no	*N*	1	1	2	
		%	4,50%	1,80%	1,90%	
	I don't know	*N*	6	17	19	
		%	27,30%	30,90%	17,60%	

Tab. 14. Relationship between respondents' education level and their attitudes toward HPVv.

			basic vocational education	secondary education	higher education	
Are you willing to undergo HPV vaccination, which can protect against HPV infection?	yes	*N*	18	42	90	$p = 0{,}452$
		%	81,80%	77,80%	84,10%	
	no	*N*	4	11	12	
		%	18,20%	20,40%	11,20%	
	już się zaszczepiłem	*N*	0	1	5	
		%	0,00%	1,90%	4,70%	
Are you willing to have your child / children vaccinated against HPV?	yes, but only my daughter(s)	*N*	7	12	21	$p < 0{,}001$<br>$V = 0{,}27$
		%	35,00%	21,80%	19,60%	
	yes, but only my son(s)	*N*	0	0	5	
		%	0,00%	0,00%	4,70%	
	yes, regardless of the sex of	*N*	7	30	74	
		%	35,00%	54,50%	69,20%	

	my child / children				
	no	*N*	5	13	4
		%	25,00%	23,60%	3,70%
	my child / children have already been vaccinated	*N*	1	0	3
		%	5,00%	0,00%	2,80%

Tab. 15. Correlation between respondents' education level and their awareness of the 50% reimbursement for HPVv.

			basic vocational education	secondary education	higher education
Are you aware that the HPV vaccine is in 50% co-funded?	yes	*N*	3	7	21
		%	13,60%	12,70%	19,40%
	no	*N*	19	48	87
		%	86,40%	87,30%	80,60%

Tab. 16. Comparison of awareness of HPV to knowledge about HPV.

			Have you heard of the human papilloma virus (HPV)?		
			yes	no	
Do you believe that HPV causes cervical cancer?	yes	*N*	125	4	$p < 0,001$ $V = 0,63$
		%	82,80%	11,10%	
	no	*N*	2	0	
		%	1,30%	0,00%	
	I don’t know	*N*	24	32	
		%	15,90%	88,90%	
Is HPV infection asymptomatic?	yes	*N*	82	2	$\chi^2(2) = 36,39$ $p < 0,001$ $V = 0,44$
		%	54,30%	5,60%	
	no	*N*	21	3	
		%	13,90%	8,30%	
	I don’t know	*N*	48	31	
		%	31,80%	86,10%	
Is HPV infection a sexually transmitted disease?	yes	*N*	115	13	$\chi^2(2) = 24,33$ $p < 0,001$ $V = 0,36$
		%	76,70%	36,10%	
	no	*N*	9	3	
		%	6,00%	8,30%	
	I don’t know	*N*	26	20	
		%	17,30%	55,60%	
	yes	*N*	124	16	

Can HPV infection cause an abnormal cytology result?		%	82,10%	44,40%	*p* < 0,001 *V* = 0,38
	no	*N*	4	0	
		%	2,60%	0,00%	
	I don’t know	*N*	23	20	
		%	15,20%	55,60%	

Tab. 17. Correlation between awareness of HPV and the willingness to undergo vaccination for oneself and vaccinate one's child/children against HPV.

			Have you heard of the human papilloma virus (HPV)?		
			yes	no	
Are you willing to undergo HPV vaccination, which can protect against HPV infection?	yes	*N*	124	28	*p* = 0,229
		%	83,20%	77,80%	
	no	*N*	19	8	
		%	12,80%	22,20%	
	I have already been vaccinated	*N*	6	0	
		%	4,00%	0,00%	
Are you willing to have your child / children vaccinated against HPV?	yes, but only my daughter(s)	*N*	31	9	*p* < 0,001 *V* = 0,40
		%	20,80%	25,70%	
	yes, but only my son(s)	*N*	4	1	
		%	2,70%	2,90%	
	yes, regardless of the sex of my child / children	*N*	101	12	
		%	67,80%	34,30%	
	no	*N*	9	13	
		%	6,00%	37,10%	
	my child / children have already been vaccinated	*N*	4	0	
		%	2,70%	0,00%	

Tab. 18. Assessment of the readiness for HPVv for oneself in comparison to the readiness to vaccinate one's child or children.

			Are you willing to undergo HPV vaccination, which can protect against HPV infection?			
			Yes	No	I have already been vaccinated	
Are you willing to have your child / children vaccinated against HPV?	Yes, but only my daughter(s)	*N*	35	3	0	*p* < 0,001 *V* = 0,44
		%	23,30%	11,50%	0,00%	
	Yes, but only my son(s)	*N*	3	2	0	
		%	2,00%	7,70%	0,00%	
		N	102	6	5	

	Yes, regardless of the sex of my child / children	%	68,00%	23,10%	83,30%	
	no	*N*	7	15	0	
		%	4,70%	57,70%	0,00%	
	My child / children have already been vaccinated	*N*	3	0	1	
		%	2,00%	0,00%	16,70%	

Tab. 19. Evaluation of knowledge about HPV in relation to the inclination to vaccinate one's child or children.

			Are you willing to have your child / children vaccinated against HPV?			
			Yes, regardless of the sex of my child / children or My child / children have already been vaccinated	Yes, but only my daughter(s) or Yes, but only my son(s)	No	
Do you believe that HPV causes cervical cancer?	yes	*N*	92	31	5	$p < 0{,}001$ $V = 0{,}29$
		%	78,60%	68,90%	22,70%	
	no	*N*	2	0	0	
		%	1,70%	0,00%	0,00%	
	I don't know	*N*	23	14	17	
		%	19,70%	31,10%	77,30%	
Is HPV infection asymptomatic?	yes	*N*	66	16	2	$\chi^2(2) = 29{,}50$ $p < 0{,}001$ $V = 0{,}28$
		%	56,40%	35,60%	9,10%	
	no	*N*	18	5	1	
		%	15,40%	11,10%	4,50%	
	I don't know	*N*	33	24	19	
		%	28,20%	53,30%	86,40%	
Is HPV infection a sexually transmitted disease?	yes	*N*	90	27	10	$p = 0{,}012$ $V = 0{,}18$
		%	76,90%	61,40%	45,50%	
	no	*N*	7	2	3	
		%	6,00%	4,50%	13,60%	
	I don't know	*N*	20	15	9	
		%	17,10%	34,10%	40,90%	
Can HPV infection cause an abnormal cytology result?	yes	*N*	92	35	11	$p = 0{,}040$
		%	78,60%	77,80%	50,00%	
	no	*N*	3	1	0	
		%	2,60%	2,20%	0,00%	

	I don’t know	*N*	22	9	11
		%	18,80%	20,00%	50,00%
